# Analysis of sexual dysfunction after abdominal aortic aneurysm repair with the type of surgical technique adopted

**DOI:** 10.1590/1677-5449.202401612

**Published:** 2025-03-14

**Authors:** Vanessa Souza Silva Medrado, Edenilson de Souza Teixeira, Hideki Zimermann Kamitani, Domingos Sávio de Oliveira e Silva, Leonardo Alves Santos, Rodrigo Mendes, Pedro Pereira Tenório

**Affiliations:** 1 Universidade Federal do Vale do São Francisco – UNIVASF, Paulo Afonso, BA, Brasil.; 2 Irmandade Santa Casa de Misericórdia de São Paulo SP, Brasil.

Dear Editor,

The study published by Schmid et al., entitled “*Sexual dysfunction after open abdominal aortic aneurysm repair: 16 years’ experience in a quaternary center and literature review*”, highlighted how surgical repair of abdominal aortic aneurysms (AAA) can cause sexual dysfunction (SD), including retrograde ejaculation and erectile dysfunction (ED), in patients treated with different aortic reconstruction techniques.^1^

The causes of SD may be psychological, organic, or pharmaceutical, and the main organic causes are of vasculogenic origin, linked to atherosclerotic lesions, cavernosa dysfunction, or veno-occlusive insufficiency. Studies such as those by Donato et al.^2^ and Meller et al.^3^ indicate that ED shares risk factors with coronary artery disease (CAD), peripheral arterial disease (PAD), and diseases of the carotid artery.^2,3^

Hypercholesterolemia, systemic arterial hypertension (SAH), smoking, and atherosclerosis contribute to vasculogenic ED.^2,4^ These factors may have influenced the results reported by Schmid et al.,^1^ since the sample analyzed included patients with SAH, dyslipidemia, smoking, and diabetes mellitus, with varied risk factors. Although the study attempted to establish causal relationships between the comorbidities, the heterogeneous nature of risk factors, including psychological factors, was not fully explored.

The single-center nature of the study limited generalization of the results, as did exclusion of patients without regular follow-up, which could have introduced selection bias. The statistical analysis conducted was adequate, but there was no adjustment for factors such as age, comorbidities, and follow-up time, which are essential to correctly interpret the greater prevalence of ED among patients with an aorto-bifemoral by-pass and of retrograde ejaculation among aorto-aortic by-pass patients. Moreover, the lack of a control group makes it difficult to interpret the results.

Notwithstanding, studies such as one by Dariane et al.^5^ suggest that endovascular aneurysm repair (EVAR) can reduce SD compared with open AAA surgery, because of reduced nerve injury and fewer changes to pelvic blood flow. The causes of SD may be disorders of excitation, orgasm, or ejaculation, ED, or vascular factors ^3-5^ The benefits of EVAR for preservation of sexual function are due to protection of nerves, but it can be compromised by complications such as fibrosis and inflammation.^5^
