# Supplementary material for: Analysis of sexual dysfunction after abdominal aortic aneurysm repair with the type of surgical technique adopted
Source: J Vasc Bras. 2025 Mar 14;24:e20240161. doi: 10.1590/1677-5449.202401612 (PMC12042766; doi:10.1590/1677-5449.202401612)
Supplement: Translation

# Translation: Análise da disfunção sexual após reparo de aneurisma de aorta abdominal com o tipo de técnica cirúrgica adotada

**DOI:** 10.1590/1677-5449.202401611

**Authors:** Vanessa Souza Silva Medrado, Edenilson de Souza Teixeira, Hideki Zimermann Kamitani, Domingos Sávio de Oliveira e Silva, Leonardo Alves Santos, Rodrigo Mendes, Pedro Pereira Tenório

**Affiliations:** 1 Universidade Federal do Vale do São Francisco – UNIVASF, Paulo Afonso, BA, Brasil.; 2 Irmandade Santa Casa de Misericórdia de São Paulo, São Paulo, SP, Brasil.

Caro Editor,

O estudo publicado por Schmid et al., intitulado “*Sexual dysfunction after open abdominal aortic aneurysm repair: 16 years’ experience in a quaternary center and literature review*”, destacou como o reparo cirúrgico do aneurisma da aorta abdominal (AAA) pode causar disfunção sexual (DS), incluindo ejaculação retrógrada e disfunção erétil (DE), em pacientes submetidos a diferentes técnicas de reconstrução aórtica^1^ .

A DS pode ter causas psicológicas, orgânicas ou medicamentosas, sendo a principal causa orgânica de origem vasculogênica, ligada a lesões ateroscleróticas, disfunção cavernosa ou insuficiência veno-oclusiva. Estudos como os de Donato et al.^2^ e Meller et al.^3^ indicaram que a DE compartilha fatores de risco com a doença arterial coronariana (DAC), a doença arterial periférica (DAP) e as doenças da artéria carótida^2,3^ .

Hipercolesterolemia, hipertensão arterial sistêmica (HAS), tabagismo e aterosclerose contribuem para a DE vasculogênica^2,4^ . Esses fatores podem ter influenciado os resultados de Schmid et al.^1^ , pois o grupo analisado incluía pacientes com HAS, dislipidemia, tabagismo e diabetes melito, com fatores de risco variados. Apesar de buscar estabelecer uma relação causal entre as comorbidades, a heterogeneidade dos fatores de risco, incluindo psicológicos, não foi completamente abordada.

A realização do estudo em um único centro limitou a generalização dos resultados, além da exclusão de pacientes sem acompanhamento regular, o que pode ter gerado viés de seleção. A análise estatística adotada foi adequada, porém faltaram ajustes para fatores como idade, comorbidades e tempo de seguimento, essenciais para interpretar corretamente a maior prevalência de DE em pacientes com *by-pass* aorto-bifemoral e ejaculação retrógrada com *by-pass* aorto-aórtico. Ademais, a ausência de um grupo-controle dificultou a interpretação dos resultados.

Outrossim, estudos como o de Dariane et al.^5^ sugerem que o reparo endovascular de aneurisma (EVAR) pode reduzir a DS em comparação à cirurgia aberta de AAA, devido a menor lesão nervosa e menores alterações no fluxo sanguíneo pélvico. A DS pode ser causada por distúrbios de excitação, orgasmo, ejaculação, DE ou fatores vasculares^3-5^ . O benefício do EVAR na preservação da função sexual deve-se à proteção dos nervos, mas complicações como fibrose e inflamação podem prejudicá-la^5^ .
