# Supplementary material for: Analysis of sexual dysfunction after abdominal aortic aneurysm repair with the type of surgical technique adopted
Source: J Vasc Bras. 2025 Mar 14;24:e20240161. doi: 10.1590/1677-5449.202401612 (PMC12042766; doi:10.1590/1677-5449.202401612)
Supplement: Related texts

# Reply Letter: sexual dysfunction after open abdominal aortic aneurysm repair: 16 years’ experience in a quaternary center and literature review

**Authors:** Bruno Pagnin Schmid, Marcelo Vezzi Muce, Rodrigo Gonzales Bocos, Fábio Hüsemann Menezes

**Affiliations:** 1 Universidade Estadual de Campinas – UNICAMP, Campinas, SP, Brasil.; 2 Hospital Israelita Albert Einstein – HIAE, São Paulo, SP, Brasil.

The authors are grateful for the comments received and honored by the opportunity to discuss the article’s findings in even greater depth.

The lack of a control group is a limitation that is intrinsic to the study design, as is recruitment of patients from just one center, as explained in the Discussion section and mentioned in the Letter to the Editor.^1^

Along similar lines, while the absence of stratification by risk factors within the groups treated with different aortic reconstruction techniques could constitute an additional limitation, arterial hypertension was observed in 99% of the patients in the study and smoking was present in 92%. As such, almost the entire sample under analysis was predisposed to arteriopathy.

Additionally, while the failure to pair for psychogenic factors involved in erectile dysfunction, as mentioned in the Letter to the Editor, could be a confounding factor worth considering, this is a less likely causal factor in a cohort of patients who have undergone open repair of abdominal aortic aneurysm, with clear anatomic interference affecting the pelvic arterial circulation and the hypogastric nerve plexus.

It should also be pointed out that the study sample included patients followed from 1995 to 2000, which was a time when the concepts of preservation of the hypogastric artery were still in development. As pointed out in the letter, other studies have ratified endovascular abdominal aortic aneurysm repair as a technique with a lower incidence of sexual dysfunction when compared to open repair and have reaffirmed the importance of preservation of the hypogastric arteries.^2-4^

Finally, we highlight the importance of discussing sexual dysfunction as a potential complication during preoperative counseling with patients who are candidates for open repair of aneurysms of the aorta. Moreover, it is also relevant to investigate the possible influence of the type of aortic reconstruction configuration on the type of sexual dysfunction observed.

# Reply: Carta Resposta: disfunção sexual após correção aberta de aneurisma da aorta abdominal: 16 anos de experiência em um centro quaternário e revisão da literatura

**Published:** 2025-02-28

**Authors:** Bruno Pagnin Schmid, Marcelo Vezzi Muce, Rodrigo Gonzales Bocos, Fábio Hüsemann Menezes

**Affiliations:** 1 Universidade Estadual de Campinas – UNICAMP, Campinas, SP, Brasil.; 2 Hospital Israelita Albert Einstein – HIAE, São Paulo, SP, Brasil.

Os autores agradecem o comentário recebido e se sentem honrados com a oportunidade de discutir os achados do artigo de forma ainda mais aprofundada.

A ausência de um grupo-controle é uma limitação intrínseca ao desenho do estudo, assim como a inclusão de pacientes de um único centro, conforme destacado na seção de Discussão e na carta ao editor^1^ .

Do mesmo modo, a ausência da estratificação dos fatores de risco entre os grupos submetidos às diferentes técnicas de reconstrução aórtica pode representar uma limitação adicional. Entretanto, entre os pacientes incluídos no estudo, a hipertensão arterial foi notada em 99% e o tabagismo, em 92%, o que contempla a quase totalidade dos indivíduos em análise como predispostos a arteriopatias.

Além disso, a falta de pareamento dos fatores psicogênicos envolvidos na disfunção erétil, conforme levantado na carta ao editor, pode ser um fator confundidor a ser considerado. Porém, trata-se de um fator causal menos provável em uma coorte de pacientes submetidos a correção aberta de aneurisma de aorta abdominal, com interferência anatômica clara na circulação arterial pélvica e no plexo nervoso hipogástrico.

Ainda cabe ressaltar que a amostra do estudo incluiu pacientes acompanhados entre 1995 e 2000, período em que os conceitos de preservação da artéria hipogástrica ainda estavam em construção. Como destacado no comentário enviado, outros estudos ratificam o reparo endovascular do aneurisma de aorta abdominal como uma técnica com menor incidência de disfunção sexual quando comparada ao reparo aberto, além de reafirmarem a importância da manutenção das artérias hipogástricas^2-4^ .

Finalmente, ressaltamos a importância da abordagem da disfunção sexual durante o aconselhamento pré-operatório como uma complicação em potencial nos pacientes candidatos ao reparo aberto do aneurisma de aorta. Além disso, é relevante investigar a possível influência do tipo de configuração da reconstrução aórtica no tipo de disfunção sexual apresentado.

## References

[1] Schmid BP, Muce MV, Bocos RG, Menezes FH (2024). Sexual dysfunction after open abdominal aortic aneurysm repair: 16 years’ experience in a quaternary center and literature review. J Vasc Bras.

[2] Fatima J, Correa MP, Mendes BC, Oderich GS (2012). Pelvic revascularization during endovascular aortic aneurysm repair. Perspect Vasc Surg Endovasc Ther.

[3] Arenas Azofra E, Iglesias Iglesias C, Canga Gonzalez C (2022). Midterm results of endovascular versus open surgical repair for infrarenal abdominal aortic aneurysms in low surgical risk patients. J Vasc Surg.

[4] Regnier P, Lareyre F, Hassen-Khodja R, Durand M, Touma J, Raffort J (2018). Sexual dysfunction after abdominal aortic aneurysm surgical repair: current knowledge and future directions. Eur J Vasc Endovasc Surg.
